# NMR Study of the Secondary Structure and Biopharmaceutical Formulation of an Active Branched Antimicrobial Peptide

**DOI:** 10.3390/molecules24234290

**Published:** 2019-11-25

**Authors:** Francesca Castiglia, Fabrizia Zevolini, Giulia Riolo, Jlenia Brunetti, Alessandra De Lazzari, Alberto Moretto, Giulia Manetto, Marco Fragai, Jenny Algotsson, Johan Evenäs, Luisa Bracci, Alessandro Pini, Chiara Falciani

**Affiliations:** 1 Department of Medical Biotechnology, University of Siena, via Aldo Moro 2, 53100 Siena, Italy; free.castiglia@gmail.com (F.C.); fabrizia.zevolini@student.unisi.it (F.Z.); giulia.riolo@gmail.com (G.R.); jlenia.brunetti@unisi.it (J.B.); luisa.bracci@unisi.it (L.B.); alessandro.pini@unisi.it (A.P.); 2 Zambon S.p.a, Via della Chimica, 9. 36100 Vicenza, Italy; Alessandra.DeLazzari@zambongroup.com (A.D.L.); Alberto.Moretto@zambongroup.com (A.M.); Giulia.Manetto@zambongroup.com (G.M.); 3 Magnetic Resonance Center (CERM), University of Florence, via Luigi Sacconi 6, 50019 Sesto Fiorentino (Firenze), Italy; fragai@cerm.unifi.it; 4 Red Glead Discovery AB, Medicon Village, 223 81 Lund, Sweden; jenny.algotsson@redglead.com (J.A.); johan.evenas@redglead.com (J.E.); 5 SetLance srl, via Aldo Moro 2, 53100 Siena, Italy

**Keywords:** antimicrobial peptides, branched peptides, NMR structure, counter-ion

## Abstract

The synthetic antimicrobial peptide SET-M33 is being developed as a possible new antibacterial candidate for the treatment of multi-drug resistant bacteria. SET-M33 is a branched peptide featuring higher resistance and bioavailability than its linear analogues. SET-M33 shows antimicrobial activity against different species of multi-resistant Gram-negative bacteria, including clinically isolated strains of *Pseudomonas aeruginosa*, *Klebsiella pneumoniae*, *Acinetobacter baumanii* and *Escherichia coli*. The secondary structure of this 40 amino acid peptide was investigated by NMR to fully characterize the product in the framework of preclinical studies. The possible presence of helixes or β-sheets in the structure had to be explored to predict the behavior of the branched peptide in solution, with a view to designing a formulation for parenteral administration. Since the final formulation of SET-M33 will be strictly defined in terms of counter-ions and additives, we also report the studies on a new salt form, SET-M33 chloride, that retains its activity against Gram-negative bacteria and gains in solubility, with a possible improvement in the pharmacokinetic profile. The opportunity of using a chloride counter-ion is very convenient from a process development point of view and did not increase the toxicity of the antimicrobial drug.

## 1. Introduction

**Figure 1:**
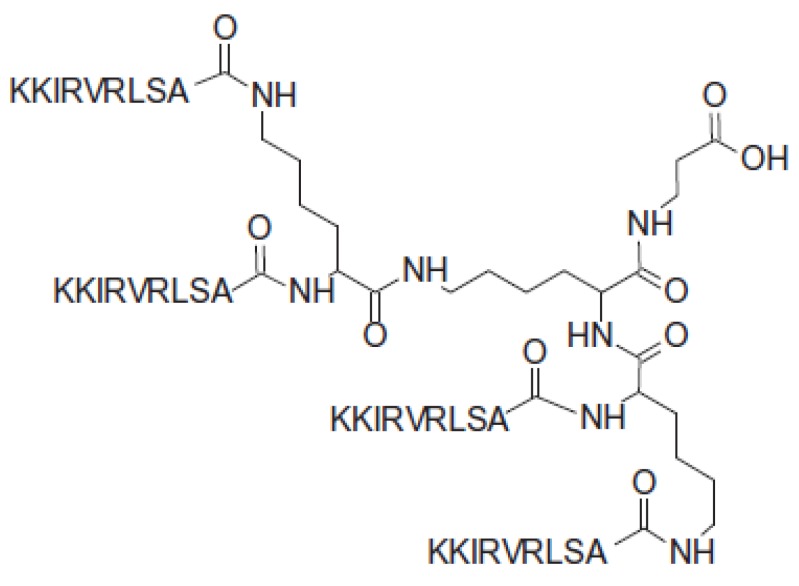
Tetra-branched structure of the antimicrobial peptide SET-M33.

The synthetic antimicrobial peptide SET-M33 is being developed as a possible new candidate for the development of a new antibacterial drug [1]. Synthesized in branched form with four copies of the same peptide sequence (KKIRVRLSA) on a three-lysine core (Figure 1), it is more resistant to proteases and more suitable for clinical applications than its linear analogues [2,3,4,5]. SET-M33 shows strong antimicrobial activity against several multi-resistant Gram-negative bacteria, including clinical isolates of *Pseudomonas aeruginosa*, *Klebsiella pneumoniae*, *Acinetobacter baumanii* and some enterobacteriaceae [6,7]. It also shows anti-inflammatory properties thanks to its capacity to neutralize LPS-induced cytokine release in vitro and in vivo and to prevent septic shock in animals infected with bacteria of clinical interest [8].

The upstream and downstream processes of SET-M33 are currently being developed together with full characterization of the product. Like most peptides, SET-M33 is routinely characterized by HPLC and mass spectrometry for its purity and chemical structure. Here we also report its ^1^H/^13^C/^15^N NMR spectroscopy characterization for full atom-specific assignment and its primary and secondary peptide structure based on observed chemical shifts. Assessment of SET-M33 secondary structure in water allows predicting aggregation potential, which can affect drug product design and formulation strategy. Aqueous environments are involved in most typical peptide drug manufacturing processes, that can be crucial for successful development, such as the lyophilization process and the preparation of injectable pharmaceutical forms.

Salt formation is important in biopharmaceutical drug development as it modulates drug solubility, stability and bioavailability. Like almost all synthetic peptides, SET-M33 is obtained as a trifluoroacetate salt (TFA), due to the presence of trifluoroacetic acid in the cleavage and purification settings. Preliminary studies on the efficacy and toxicity of SET-M33 revealed that SET-M33 acetate is less toxic to human cells and animals than SET-M33 TFA [9]. The SET-M33 peptide is therefore converted to the acetate form at the end of synthesis. The counter-ion exchange requires the use of a quaternary ammonium salt resin and is unsuitable for industrial scaling [10]. In order to avoid this step, hydrochloric acid was chosen as a stronger acid than TFA, so that the chloride counter-ion could replace trifluoroacetate [11,12,13]. Complete replacement of TFA with chloride and stability of SET-M33 to HCl treatment were assessed. Finally, the antimicrobial activity and toxicity of SET-M33 with the three different counter-ions, TFA, acetate and chloride, were assessed in mammalian cells and in mice.

## 2. Results and Discussion

### 2.1. NMR Characterization of SET-M33 Peptide

The 40 amino acid long peptide SET-M33 was characterized using ^1^H-/^13^C-/^15^N-NMR spectroscopy to perform complete atom-specific assignment, to verify the primary peptide structure and to evaluate possible secondary or other ordered structures based on observed chemical shift values and possible medium- and long-range NOEs.

**Figure 2:**
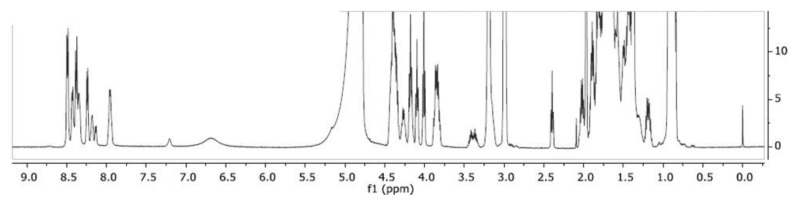
1D ^1^H-NMR spectrum of 12.5 mg/mL SET-M33 in H_2_O/D_2_O (9:1) and TFA-*d* (12.5 mg/mL) at 298 K.

To allow a faster NMR data collection, a 12.5 mg/mL peptide solution was used. The pH was adjusted to pH 4.9 by a small addition of TFA-*d* in order to minimize the rate of proton exchange between amide groups of the peptide and water molecules resulting in clear observation of the backbone amide proton signals critical to effectively obtain the atom-specific chemical shift assignments. The resulting 1D ^1^H-NMR spectrum is shown in Figure 2.

**Figure 3:**
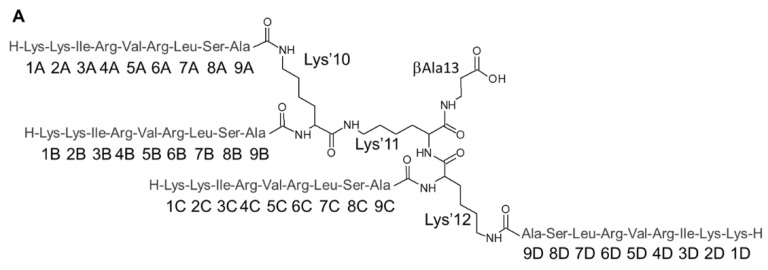

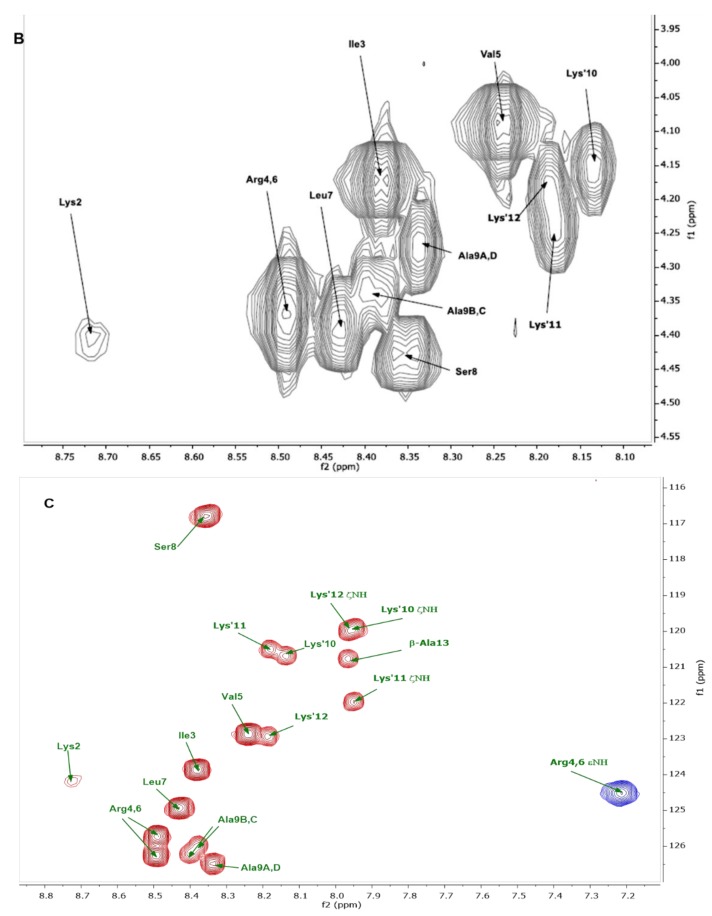
(**A**) The primary structure of SET-M33 with amino acid numbering. Amino acids are labeled with a three-letter code and sequence number as reported above. (**B**) The ^1^HN-^1^Hα fingerprint region of the 2D ^1^H COSY spectrum of SET-M33 in H_2_O/D_2_O (9:1) with addition of TFA-*d*, pH 4.9 at 298 K. The assignment of each cross-peak is shown using the three-letter amino acid code followed by the sequence number. (**C**) The 2D ^1^H-^15^N HSQC spectrum of SET-M33 in H_2_O/D_2_O (9:1) with addition of TFA-*d* at 298 K. The peaks for the sidechain εNH groups of Arg4,6 are folded into the spectrum with opposite sign (blue color). The assignment of each cross-peak is indicated using the three-letter amino acid code followed by the sequence number.

The spectral quality and resolution were good although the amide region contained some broadened signals, presumably due to fast proton exchange with the solvent. All observed signals originated from SET-M33, except those that originate from residual NMR solvents and DSS. In addition, a singlet ^1^H signal was observed at 1.97 ppm (proton(s) bound to carbon with a ^13^C chemical shift of 25.0 ppm), tentatively assigned to acetate. The broad ^1^H signal at 6.68 ppm sharpened further at pH values lower than 4.9, and an additional broad peak also appeared at 7.54 ppm at these more acidic conditions (data not shown). These two broad signals presumably originated from the guanidine- and amino side chain groups of arginine and lysine residues. The first step in the NMR study was to assign the ^1^H, ^13^C and ^15^N chemical shifts of SET-M33 applying the conventional backbone amide proton strategy developed for non-isotope-enriched proteins [14]. Figure 3 (panel **A**) shows the amino acid numbering and atom labelling of SET-M33. The four identical segments of nine amino acids are indicated A, B, C and D. The three linking lysine residues are indicated Lys’10, Lys’11 and Lys’12, respectively and the C-terminal βAla, as βAla 13.

The ^1^HN-^1^Hα fingerprint region of the 2D ^1^H COSY spectrum and the ^1^HN-^15^N fingerprint region of the ^1^H-^15^N HSQC spectrum are shown in Figure 3 (panels B and C, respectively), with each peak labelled with the assigned amino acid. The amino group of Lys1 was not detectable due to fast proton exchange with the solvent and also the backbone amide proton signal of Lys2 was significantly weaker presumably for the same reason. The assignment procedure readily revealed virtually identical chemical shifts of the four 9-amino acid chains, strongly indicating the same conformation(s) of all the branches of SET-M33. There were, however, small chemical shift changes clearly observed between Ala9 of A,D and Ala9 of B,C, consistent with the asymmetric coupling to the branched core, i.e., A,D are connected via the sidechain amino group of Lys’10 and Lys’12 while B,C are connected via the backbone amino groups of Lys’10 and Lys’12.

**Figure 4:**
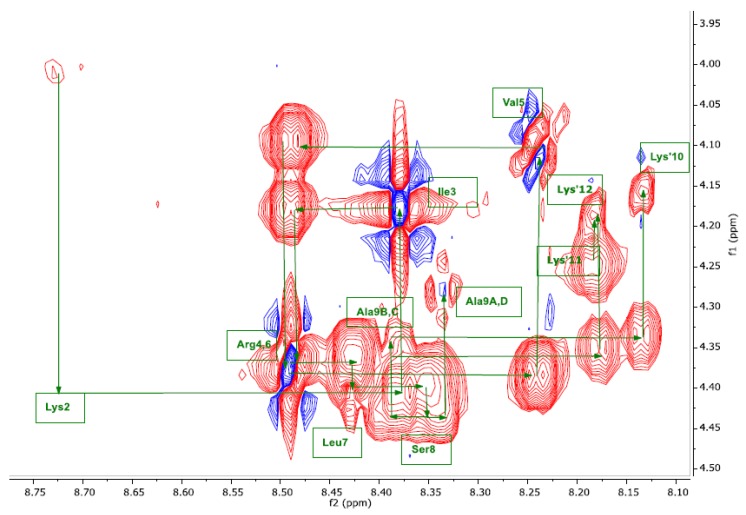
Sequential walk through the amide backbone fingerprint region of 2D ^1^H NOESY spectrum of SET-M33 in H_2_O/D_2_O (9:1) with addition of TFA-*d*, pH 4.9 298 K is shown. The assignment of each cross-peak is shown using the three-letter amino acid code followed by the sequence number. The sequential walk follows the arrows between alternating sequential inter-residue HN(i)-Hα(i-1) and intra-residue HN(i)-Hα(i) NOEs.

The primary structure was confirmed by the “sequential walk” in the ^1^HN(i)-^1^Hα fingerprint region of the 2D ^1^H NOESY spectrum connecting sequential amino acids (Figure 4). This plot showed the “walk” between alternating sequential and intra-residue NOE (^1^HN-^1^Hα) from Lys1 to Lys’10, Lys’11 and Lys’12 – a key step to confirm the amino acid sequence. All assigned ^1^H-, ^13^C-, and ^15^N-NMR chemical shifts were reported in Table 1 and fully confirmed the anticipated amino acid sequence and chemical structure of SET-M33.

The chemical shifts are determined in the 2D ^1^H-^13^C HSQC and 2D ^1^H-^15^N HSQC spectra. Referencing for ^1^H- and ^13^C- spectra is relative to the ^1^H- and ^13^C-NMR chemical shifts of the methyl groups of DSS (set to 0 ppm for both nuclei) while indirect chemical shift referencing is applied for ^15^N chemical shifts using the gyromagnetic ratio of ^1^H and ^15^N. The notation *NO* indicates that the signals of these atoms are not observed. The ^15^N-NMR chemical shifts of Arg4 and Arg6 may be interchanged, i.e., the ^15^N chemical shift of Arg4 may belong to Arg6 and vice versa.

Based on the peak integral measured of aliphatic protons in the quantitative 1D ^1^H-NMR spectrum, the number of amino acids in the peptide was verified.

### 2.2. Assessment of Secondary Structure Elements

**Figure 5:**
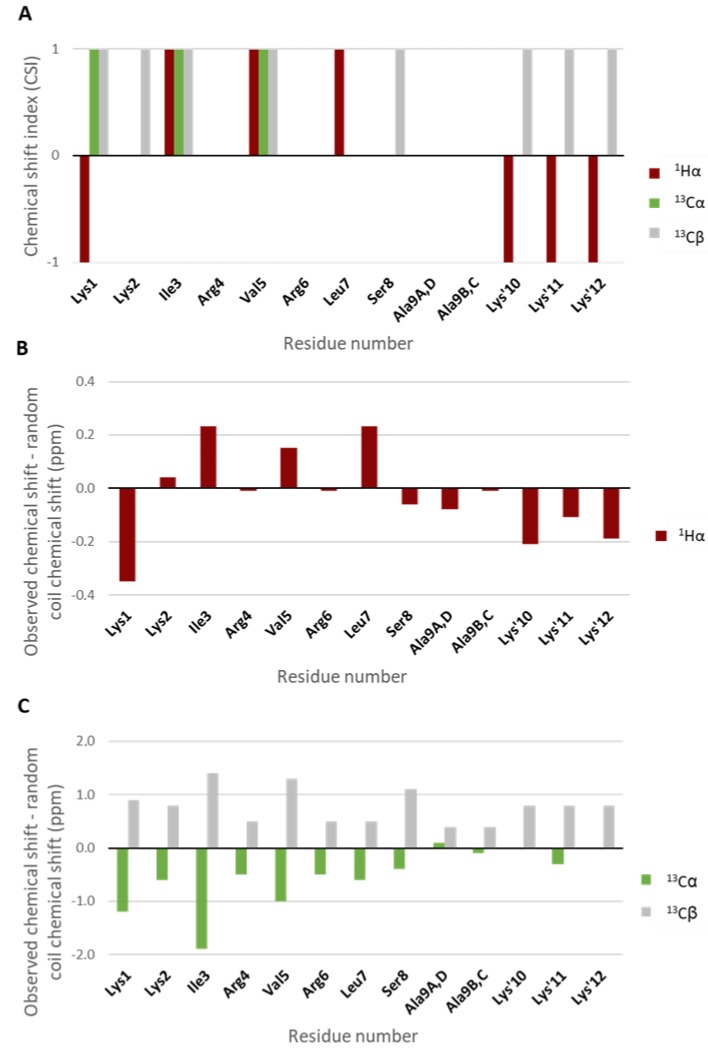
^1^Hα, ^13^Cα and ^13^Cβ chemical shift data of SET-M33 in H_2_O/D_2_O (9:1) with addition of TFA-*d*, pH 4.9 at 298 K. (**A**) CSI values for ^1^Hα, ^13^Cα and ^13^Cβ atoms. (**B**) The absolute difference between observed chemical shift values and random coil chemical shift values for ^1^Hα atoms. (**C**) The absolute difference between observed chemical shift values and random coil chemical shift values for ^13^Cα and ^13^Cβ atoms.

The chemical shift index (CSI) method for ^1^Hα, ^13^Cα and ^13^Cβ atoms was used as the primary means to assess the degree of secondary structure and its possible sequence-specific locations [15]. The chemical shifts obtained experimentally were compared with the so-called random coil chemical shifts of the corresponding amino acid residue type (Xxx) measured in Gly-Gly-Xxx-Ala-Gly-Gly peptides in 1M urea [16]. Figure 5 shows the CSI values for ^1^Hα, ^13^Cα and ^13^Cβ atoms, as well as the absolute deviation in ppm from the random coil shifts.

An index of 1 for more than three residues in a row indicates β-strand while an index of -1 for at least four consecutive residues indicates helical structure. The index is set to 1 if the difference between experimental shift and random coil shift is greater than 0.1 ppm (^1^Hα), greater than 0.7 ppm (^13^Cβ) or less than −0.7 ppm for ^13^Cα, while it is set at -1 if the difference is less than −0.1 ppm (^1^Hα), less than −0.7 ppm (^13^Cβ) or greater than 0.7 ppm for ^13^Cα. If the absolute chemical shift difference is less than 0.1 ppm (^1^H) or 0.7 ppm (^13^C), the index is set to 0.

The chemical shift data including the several positive CSI values of the ^1^Hα, ^13^Cα and ^13^Cβ atoms indicate a generally more extended peptide backbone configuration for several amino acids in the Lys1–Ser8 sequence. However, as there are no clear stretches of three such amino acids in a row, the conclusion is that the peptide has no stable β-strand configurations.

In addition to the chemical shift analysis, the NOESY spectra were examined to further investigate the structural properties of SET-M33 in water, pH 4.9. Besides several intra- and sequential NOEs, no resolved medium- or long-range NOEs are observed, strongly indicating the lack of any stable secondary or tertiary structure. Several sequential ^1^HN-^1^HN NOEs are observed primarily for the inner core of the peptide.

The finding of virtually identical chemical shifts for all four branches, reported above, prevents most conclusions using inter-branch NOEs as such potential cross peaks would fully overlap with the intra-branch NOE cross peaks. In conclusion, the NMR data are fully consistent with anticipated chemical structure of SET-M33 but no stable secondary or tertiary structure can be shown. Furthermore, the data show that all four 9-amino acid chains of the branched peptide are in the identical conformation(s). The data suggest that these chains have a propensity to adopt an extended, flexible conformation as compared with the so-called random coil conformation.

### 2.3. TFA/Chloride Counter Ion Exchange

SET-M33 TFA (2 mg/mL) was dissolved in 100 mM HCl solution, frozen and dried. The peptide was intact after the ion exchange procedure. The HPLC profile was unchanged and the MS profile showed a single peak of molecular mass of 4683 Da, in line with the calculated mass of C_209_H_399_N_75_O_45_.

Residual TFA counter-ions was determined by comparing the intensity of the fluorine signal in the NMR spectra of the sample before and after the exchange procedure, using the same concentration and volume of SET-M33 TFA and SET-M33 chloride and the same NMR settings. The NMR experiments were recorded at 298 K on a Bruker Avance III spectrometer operating at 800 MHz and equipped with a 5 mm PHTXI ^1^H-^13^C/^15^N probe, tuned on ^19^F. The intensity of the fluorine signal after exchange decreased by a factor of about 200 with respect to the signal recorded on the sample before exchange. The solubility of SET-M33 chloride in water was much higher (100 mg/mL) than that of the acetate (15 mg/mL) and TFA forms (10 mg/mL).

### 2.4. MIC Determination

Minimum inhibitory concentrations (MICs) of SET-M33 chloride were determined against two Gram-negative pathogens, *E. coli* TG-1 and *P. aeruginosa* PAO-1 (Table 2), and compared with SET-M33 acetate. SET-M33 chloride retained its activity against both species and actually improved from 3.0 μM to 1.5 μM against *P. aeruginosa*.

### 2.5. Toxicity of Different SET-M33 Forms for Human Cells

T24 human bladder epithelial cells, 16HBE14o- human bronchial epithelial cells and RAW264.7 mouse macrophages were used to test the cytotoxicity of SET-M33 chloride and to compare it with that of the TFA and acetate salts.

Bladder cells and human bronchial cell lines were selected with a view to using SET-M33 as local treatment for urinary tract infections and pneumonia. Being a cationic hydrophilic molecule, it is also most probably eliminated in the urine when administered systemically, as observed in previous studies [7]. Hence bladder cells will be the most targeted.

**Figure 6:**
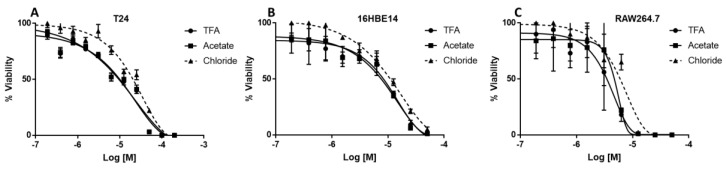
Cytotoxicity of SET-M33 salts for T24 (**A**), 16HBE14o- (**B**) and RAW264.7 (**C**) cells. Circles indicate incubation with SET-M33 TFA; squares indicate incubation with SET-M33 acetate; triangles indicate incubation with SET-M33-chloride (dotted line).

No SET-M33 form was toxic, except at very high concentrations. 100% cytotoxicity occurred at 100 μM for T24 and 16HBE14o- and at 10 μM for RAW264.7. SET-M33 chloride showed slightly lower toxicity for T24 and RAW264.7 cells (Figure 6, Table 3).

### 2.6. Acute Toxicity in Mice

Acute toxicity of SET-M33 chloride was tested in mice and compared with the acetate and TFA counter-ions. Ten animals per group (five males and five females) were injected with two doses of SET-M33 chloride, 20 and 30 mg/kg, known to be below the LD50 [7]. Signs of toxicity were scored as not-observable, mild and severe. SET-M33 chloride and SET-M33 acetate scored 100% not-observable (Table 4), unlike the TFA that scored 100% toxicity at 25 mg/kg, described as 33% severe and 66% mild [7].

## 3. Discussion

SET-M33 peptide is currently being evaluated in a late preclinical setting for its activity as an antimicrobial agent in lung infections and sepsis caused by multi-drug resistant Gram-negative bacteria [1,5,6,7]. This peptide is bactericidal and neutralizes lipopolysaccharide toxin, preventing septic shock in vivo and is therefore eligible for development as a new antibacterial drug [6]. SET-M33 is a branched peptide, composed of 40 amino acids, grouped in branches of 9-mers on a three-lysine scaffold. Its mechanism of action is based on multistep interference with bacterial membranes [17]. First it is attracted to the bacterial surface by the anionic LPS coating. Close to the lipid double layer it takes on amphipathic helix structure and becomes partially embedded in the bacterial plasma membrane, causing loss of membrane function [17]. Previous CD studies on SET-M33 and on a monomeric analogue, showed that helix formation is only observed in the presence of micelle-like structures [17].

NMR studies of multiple branched peptides are extremely uncommon because their spectra are complex due to non-equivalent branches carrying the same amino acid sequence. The secondary structure of branched peptides in solution is therefore deducted from analogue linear peptides or studied using circular dichroism techniques [17]. The branched core, forcing the oligomers into parallel distribution, may induce a beta-sheet secondary structure, that in turn could generate unwanted aggregation. It was therefore important to determine the secondary structure of SET-M33 in order to: 1) confirm the results obtained with a monomeric analogue [17], showing that a helix is only formed in the presence of micelles; 2) to investigate if a β-sheet forms in aqueous solution, so to prepare a stable drug formulation. No stable β-strand configuration of the peptide was revealed according with the chemical shift index method [15] nor supported by the NOESY data.

In developing a medicinal product, salt formulations are important. Most synthetic peptides are obtained using Fmoc–solid-phase procedures and are produced as TFA salts due to cleavage and purification conditions. TFA can be toxic for some eukaryotic cells and should therefore be removed from synthetic peptides [18,19]. TFA counter-ion is often removed using ion exchange resins in a rather lengthy procedure involving some risk of lowering peptide yield, or pKa of the peptide permitting, by repeated addition of acetic acid alternating with freeze drying [11]. Here we described a simple procedure to prepare the chloride salt of a basic peptide in quantitative yield. The complete exchange of TFA for chloride was confirmed by ^19^F-NMR. The solubility of SET-M33 chloride improved seven-fold with respect to the acetate form.

The antimicrobial efficacy of SET-M33 chloride proved to be the same as that of the acetate, actually slightly better against *P. aeruginosa*, as shown by MICs. The toxicity of the peptide was less than that of the TFA for mammalian cells and live mice. In mice, toxicity was similar to that of the acetate form, i.e., lower than the TFA. The opportunity of using a chloride counter ion is very convenient from a process development point of view. Besides, the unexpected large gain in solubility, almost seven times better than the acetate, could give the drug better pharmacokinetic features, making the new form of SET-M33 very promising.

## 4. Materials and Methods

### 4.1. M33 Peptide Synthesis

SET-M33 peptide was synthesized by solid phase synthesis in an automated Syro multiple peptide synthesizer (MultiSynTech, Witten, Germany), using standard 9-fluorenylmethyl-oxycarbonyl (Fmoc) strategy. The amino acid sequences were build up stepwise on TentaGel 4 branch β-Ala resin (loading 0.45 mmol/g) (Rapp Polymere, Tübingen, Germany) by successive cycles of Fmoc deprotection with piperidine 40%/dimethylformamide and coupling of the subsequent amino acids by activation of the respective carboxylic groups with *O*-(benzotriazol-1-yl)-*N*,*N*,*N*,*N*-tetramethyl-uronium hexafluorophosphate (HBTU)/1,3-isopropylethylamine (DIPEA). Amino acids were coupled in dimethylformamide (DMF) and the molar ratio of amino acids/HBTU/DIPEA on the resin was 5 eq/5 eq/10 eq. Side chain-protecting groups were 2,2,4,6,7-pentamethyldihydrobenzofuran-5-sulfonyl for R, t-butoxycarbonyl for K and t-butyl for S (Iris Biotech, Marktredwitz, Germany). The peptide was cleaved from the solid support, deprotected in a single step by treatment with TFA containing triisopropylsilane and water (95/2.5/2.5), and precipitated with diethyl ether. Crude peptide was purified by reverse-phase chromatography (RP-HPLC) on a Waters XBridge^®^ Peptide BEH C18 OBD Prep column (Waters, Milford, MA, USA, 300 Å, 10 μm, 19 × 250 mm), using 0.1% TFA/water as eluent A and acetonitrile as eluent B, performing a linear gradient from 83:17 A/B to 70:30 A/B in 40 min. The peptide was obtained as TFA salt. Final peptide purity was confirmed by reverse-phase chromatography on a Jupiter C18 analytical column (Phenomenex, Torrance, CA, USA, 300 Å, 5 μm, 4.6 × 250 mm), under the same conditions as for the RP-HPLC purification, and its identity was checked by MALDI ToF/ToF mass spectrometry (Ultraflex III, Brucker Daltonics, Bremen, Germany).

### 4.2. NMR

A 500 MHz Inova spectrometer (Varian, Palo Alto, CA, USA) equipped with a 5 mm ^1^H/^13^C/^15^N triple resonance probe was used for the NMR experiments. A ^1^H 90° pulse length of 10.9 μs was determined. Suppression of the intense water signal in the performed 1D and 2D NMR experiments was achieved using pre-saturation or watergate pulse sequences. A relaxation delay of 57 *s* was applied for the acquired quantitative 1D ^1^H experiments.

The following NMR spectra were recorded: 1D ^1^H ; 2D ^1^H gradient-COSY (pulsed field gradient COSY acquired in magnitude mode, standard version in VNMRJ 2.3); 2D ^1^H TOCSY (standard version in VNMRJ 2.3; mixing time = 80 ms); 2D ^1^H NOESY (standard version in VNMRJ 2.3; mixing times: 100, 200 and 400 ms); 2D ^1^H-^13^C multiplicity-edited HSQC (standard version in VNMRJ 2.3); 2D ^1^H-^15^N HSQC [20].

All spectra were recorded at 298 K with 12.5 mg/mL SET-M33 dissolved in H_2_O/D_2_O 9:1 with 0.5 μL TFA-*d* (99.5%D; Cambridge Isotope Laboratories, Tewksbury, MA, USA) with 5 μL 10 mM DSS (4,4-dimethyl-4-silapentane-1-sulfonic acid, 97%, Cambridge Isotope Laboratories) in D_2_O added as the internal chemical shift calibration standard. NMR data were processed and analyzed using MestReNova 12.0.1–20560 (Mestrelab Research SL, Santiago de Compostela, Spain). All recorded NMR spectra are presented in Supplementary Material.

### 4.3. TFA/Acetate Ion-exchange

Ion exchange from TFA to acetate was carried out using a quaternary ammonium resin in acetate form (AG1-X8, 100-200 mesh, 1.2 meq/mL capacity, BioRad, Hercules, CA, USA), with a resin: peptide ratio of 2000:1. Resin and peptide were stirred for 1 h, the resin was then filtered off and washed thoroughly. The peptide was recovered and freeze-dried.

### 4.4. TFA/Chloride Ion-exchange

SET-M33 in TFA form was dissolved in double distilled H_2_O at a concentration of 2 mg/mL (3.4 × 10^−4^ M) and mixed 1:1 with an HCl solution to yield a final HCl concentration of 50 mM. The sample was freeze dried overnight and then weighted to assess no loss of product (<95% of the calculated yield). Quantitative ^19^F-NMR and ^1^H-NMR spectra were recorded to determine the exact amount of counter ion [21,22]. The NMR experiments were performed at 298 K on an Avance III spectrometer (Bruker, Billerica, MA, USA) operating at 800 MHz (proton Larmor frequency) equipped with a 5 mm PHTXI ^1^H-^13^C/^15^N probe, tuned to ^19^F. The two samples, SET-M33 TFA and SET-M33 chloride, were prepared at the same concentration (1 mM) in 600 µL D_2_O and the spectra were acquired with 512 scans, with receiver gain factor of 64 and a relaxation delay of 6.5 s.

### 4.5. Antimicrobial Susceptibility of Bacterial Isolates – MIC Assay

Antimicrobial susceptibility was assessed by determining the minimum inhibitory concentrations (MICs) of SET-M33 acetate and SET-M33 chloride against *E. coli* TG-1 and *P. aeruginosa* PAO-1, using the broth microdilution technique according to 2017 EUCAST guidelines. The MIC assay measures visible inhibition of bacterial growth after 24 h exposure of bacteria to antibiotic in Mueller-Hinton (MH) broth. Assays were performed in triplicate using a bacterial inoculum of 5 × 10^4^ CFU/well in a final volume of 100 μL. The twofold antibiotic concentration ranged from 0.1 μM to 12 μM for both peptides. MICs were read after 24 h of incubation at 35 °C.

### 4.6. Cytotoxicity

T24 human epithelial bladder cells, 16HBE14o- human bronchial epithelial cells and RAW264.7 mouse macrophages were plated at a density of 2.5 × 10^4^ cells/well in 96-well microplates. The 16HBE14o- cells were previously incubated with coating solution (88% LHC basal medium, 10% bovine serum albumin, 30 µg/mL bovine collagen type I and 1% human fibronectin). Different concentrations of SET-M33 TFA, acetate and chloride were added 24 h after plating. Cells were grown for 48 h at 37 °C. Their viability was assessed by 3-(4,5-dimethylthiazol-2-yl)2,5-diphenyltetrazolium bromide (MTT) assay. EC50 values were obtained by dose-response variable slope analysis using GraphPad Prism 5.03 software (San Diego, CA, USA).

### 4.7. Acute Toxicity

All animal experiments were conducted under the protocol approved by the Italian Ministry of Health at the Toscana Life Sciences Foundation animal facility in Siena, Italy (authorization n. 34/2016PR). Animals (10 CD1 mice/group, 20–22 g) were injected i.v. with SET-M33 acetate (group 1 and group 2) and SET-M33 chloride (group 3 and group 4), in a single dose. The doses were: group 1, 25 mg/kg SET-M33 acetate; group 2, 30 mg/kg SET-M33 acetate; group 3, 20 mg/kg SET-M33 chloride and group 4, 30 mg/kg SET-M33 chloride. Animals were monitored for 96 h. Signs of toxicity were checked four times a day by visual inspection and were scored as not-observable, mild and severe. Mice were weighed every day from arrival to the last day of the experiment. Animals with clear signs of distress, such as reduced mobility and weight loss greater than 20%, were anesthetized and sacrificed with carbon dioxide or by cervical dislocation.

## Figures and Tables

**Table 1:** ^1^H/^13^C/^15^N chemical shifts (ppm) of SET-M33 in H_2_O/D_2_O (9:1) with addition of TFA-*d* at 298 K.

Residue	^1^HN	^15^N	^1^Hα	^13^Cα	^1^Hβ	^13^Cβ	Others
Lys1A-D	*NO*	*NO*	4.01	55.5	1.89	33.2	γ: 1.43/23.8; δ: 1.69/29.1; ε: 2.99/41.9
Lys2A-D	8.72	124.2	4.40	56.1	1.76	33.1	γ: 1.40/24.7; δ: 1.40/24.7; ε: 2.99/41.9
Ile3A-D	8.38	123.9	4.18	60.7	1.83	38.9	γ1: 1.19, 1.47/27.1; γ2: 0.89/17.3; δ1: 0.86/12.6
Arg4A-D	8.49	125.7^1^	4.37	55.8	1.75, 1.82	30.8	γ:1.58, 1.64/27.0; δ: 3.20/43.3; ε: 7.21/84.5 (^15^N)
Val5A-D	8.24	122.9	4.10	62.0	2.03	33.0	γ1: 0.92/20.8; γ2:0.94/20.5
Arg6A-D	8.49	126.3^1^	4.37	55.8	1.75, 1.82	30.8	γ:1.58, 1.64/27.0; δ: 3.20/43.3; ε: 7.21/84.5 (^15^N)
Leu7A-D	8.43	124.9	4.40	55.1	1.59, 1.64	42.4	γ: 1.64/26.8; δ1: 0.93/24.8; δ2: 0.87/23.3
Ser8A-D	8.35	116.8	4.44	57.9	3.85	63.8	
Ala9A,D	8.34	126.5	4.27	52.6	1.38	19.4	
Ala9B,C	8.40, 8.38	126.2, 126.0	4.34	52.4	1.38	19.4	
Lys’10	8.13	120.7	4.15	56.7	1.76	33.1	γ: 1.32/25.0; δ: 1.50/30.5; ε: 3.18/41.8; ζ: 7.95/119.9 (^15^N)
Lys’11	8.18	120.5	4.25	56.4	1.76	33.1	γ: 1.32/25.0; δ: 1.50/30.5; ε: 3.18/41.8; ζ: 7.95/122.0 (^15^N)
Lys’12	8.19	122.9	4.17	56.7	1.76	33.1	γ: 1.32, 1.40/25.0; δ: 1.50/30.5; ε: 3.18/41.8; ζ: 7.95/119.9 (^15^N)
β-Ala13	7.96	120.8	3.38	39.3	2.40	39.0	

**Table 2:** MICs of SET-M33 acetate and SET-M33 chloride.

Bacterial Species	MIC (μM) SET-M33	
	Acetate	Chloride
*E. coli* TG-1	1.5	1.5
*P. aeruginosa* PAO-1	3	1.5

**Table 3:** In vitro toxicity of the different SET-M33 salts, measured as inhibition of growth.

SET-M33	EC50 [M]		
Counter ion	T24	16HBE14o-	RAW264.7
TFA	1.363 × 10^−5^	1.105 × 10^−5^	3.846 × 10^−6^
Acetate	1.243 × 10^−5^	9.618 × 10^−6^	5.104 × 10^−6^
Chloride	2.260 × 10^−5^	1.034 × 10^−5^	6.125 × 10^−6^

**Table 4:** Signs of acute toxicity in vivo of the three SET-M33 salts.

SET-M33	20 mg/kg	25 mg/kg	30 mg/kg
Counter ion			
TFA	(-)	mild to severe	(-)
Acetate	not-observable [6]	not-observable	not-observable
Chloride	not-observable	(-)	not-observable

(-) not measured.

## Supplementary Materials

The following are available online, Figure S1: 1D ^1^H spectrum. Figure S2: 1D ^1^H spectrum, aliphatic region. Figure S3: 1D ^1^H spectrum, ^1^HN region. Figure S4: 2D ^1^H-^13^C multiplicity edited HSQC. Figure S5: 2D ^1^H-^15^N HSQC. Figure S6: 2D ^1^H COSY. Figure S7: 2D ^1^H COSY, aliphatic region. Figure S8: 2D ^1^H COSY, ^1^HN-^1^Hα fingerprint region. Figure S9: 2D ^1^H TOCSY with 80 ms mixing time. Figure S10: 2D ^1^H TOCSY with 80 ms mixing time, aliphatic region. Figure S11: 2D ^1^H TOCSY with 80 ms mixing time, ^1^HN region. Figure S12: 2D ^1^H NOESY with 100 ms mixing time. Figure S13: 2D ^1^H NOESY with 100 ms mixing time, aliphatic region. Figure S14: 2D ^1^H NOESY with 100 ms mixing time, ^1^HN region. Figure S15: 2D ^1^H NOESY with 200 ms mixing time. Figure S16: 2D ^1^H NOESY with 200 ms mixing time, aliphatic region. Figure S17: 2D ^1^H NOESY with 200 ms mixing time, ^1^HN region. Figure S18: 2D ^1^H NOESY with 400 ms mixing time. Figure S19: 2D ^1^H NOESY with 400 ms mixing time, aliphatic region. Figure S20: 2D ^1^H NOESY with 400 ms mixing time, ^1^HN region. Figure S21: A) 2D ^1^H TOCSY with 80 ms mixing time; B) 2D ^1^H NOESY with 200 ms mixing time
