# Supplementary material for: NMR Study of the Secondary Structure and Biopharmaceutical Formulation of an Active Branched Antimicrobial Peptide
Source: Molecules. 2019 Nov 25;24(23):4290. doi: 10.3390/molecules24234290 (PMC6930567; doi:10.3390/molecules24234290)
Supplement: Supplementary file 1 [file molecules-24-04290-s001.pdf]

Supplementary Material

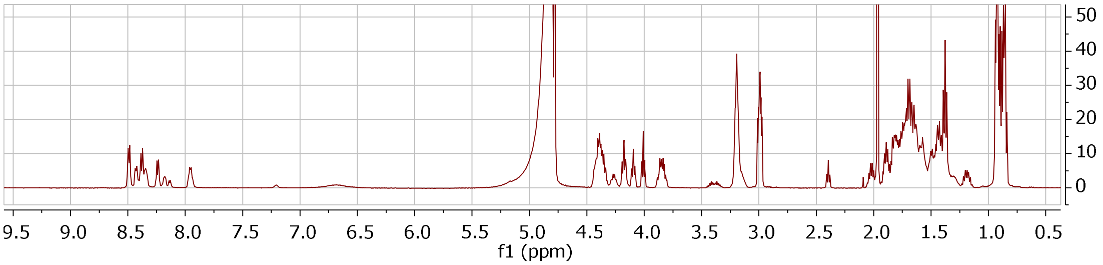

Figure S1. 1D ^{1}H spectrum

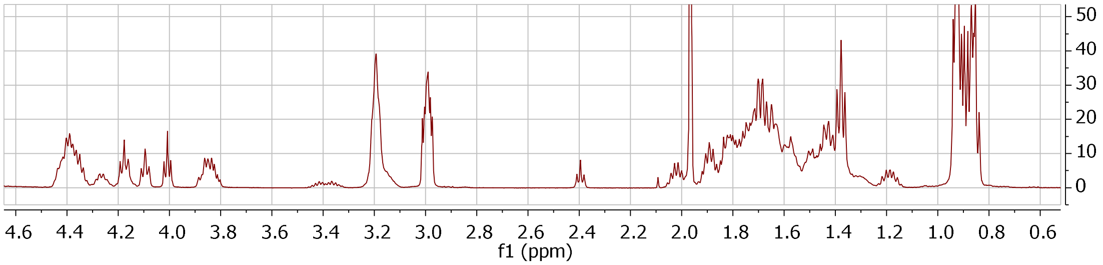

Figure S2. 1D ^{1}H spectrum, aliphatic region

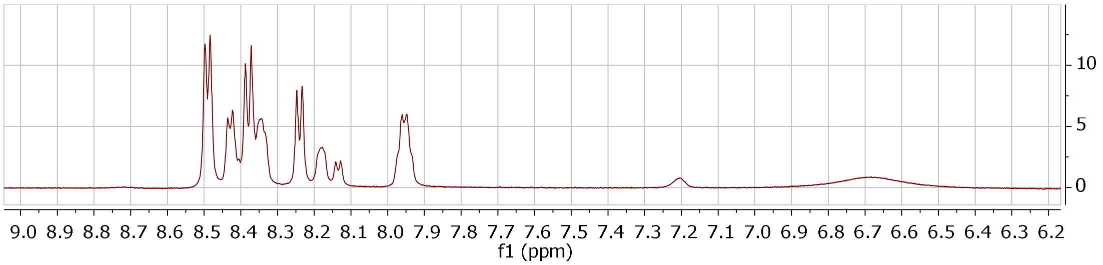

Figure S3. 1D ^{1}H spectrum, ^{1}HN region

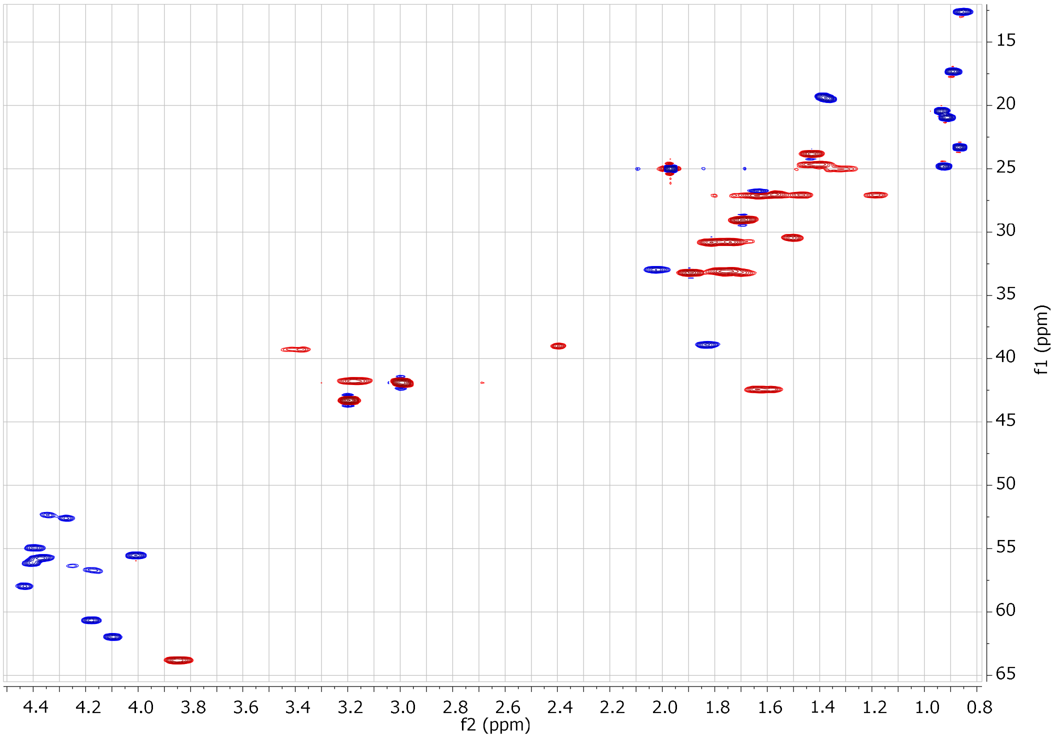

Figure S4. 2D ^{1}H-^{13}C multiplicity edited HSQC

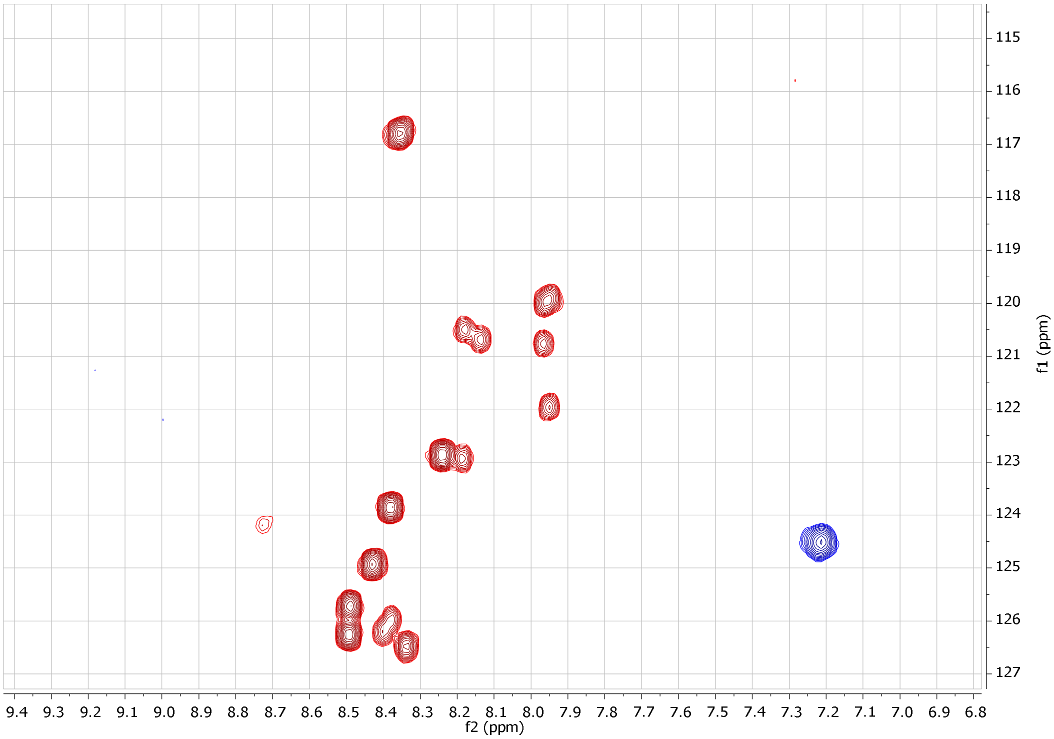

Figure S5. 2D ^{1}H-^{15}N HSQC

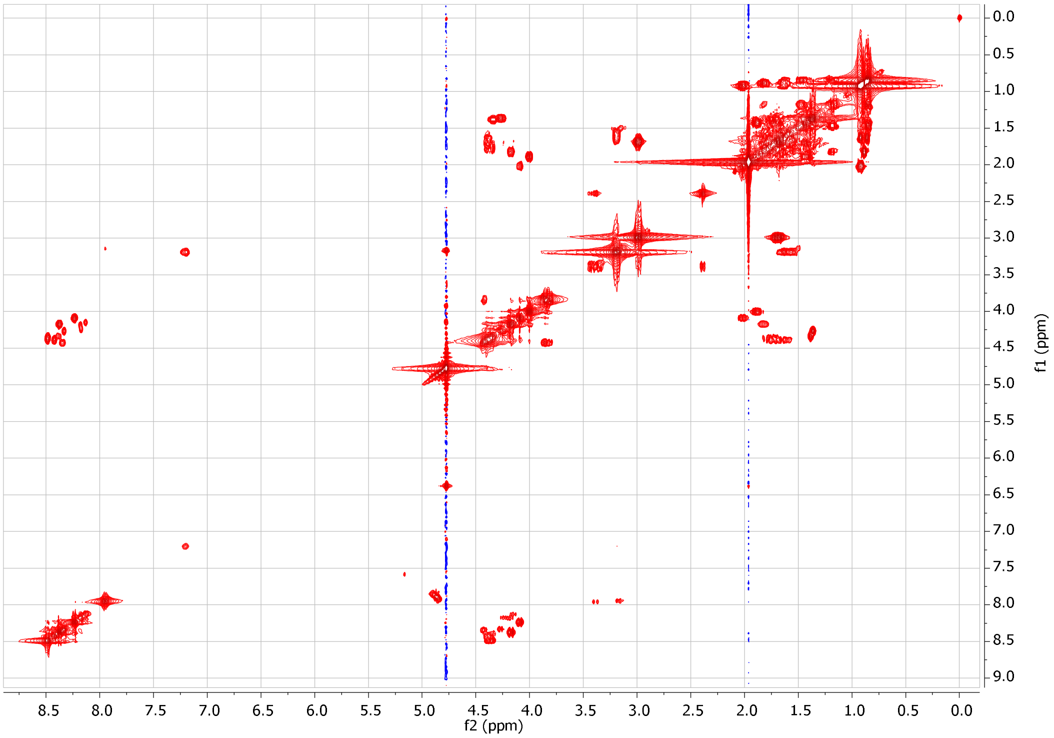

Figure S6. 2D ^{1}H COSY

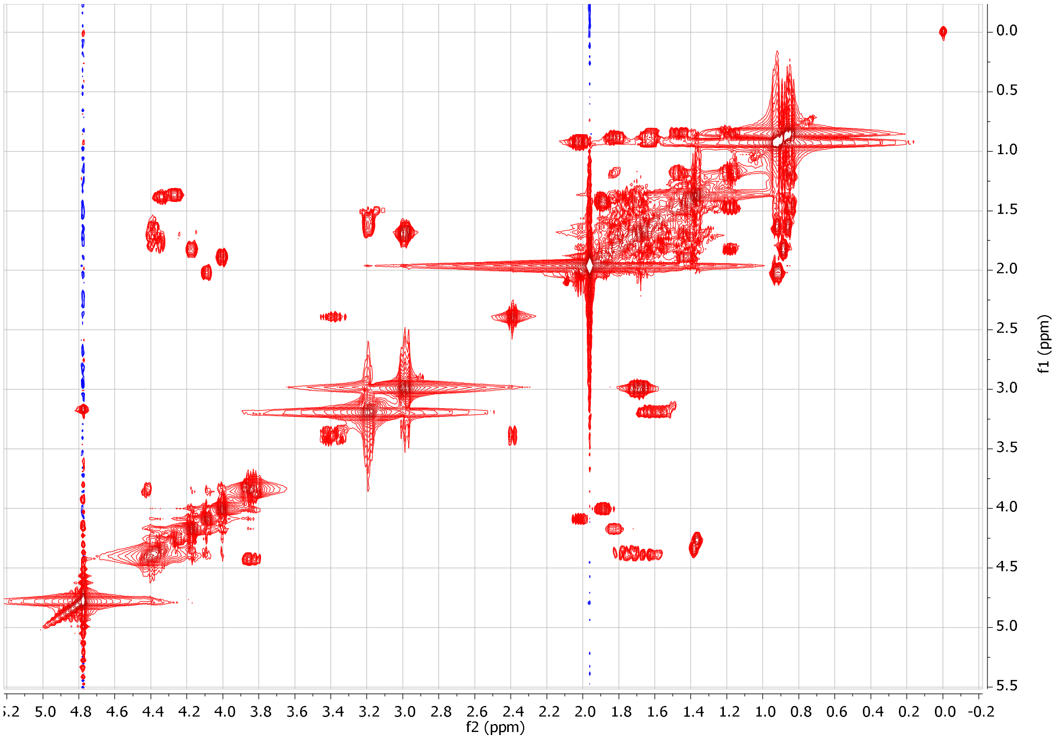

Figure S7. 2D ^{1}H COSY, aliphatic region

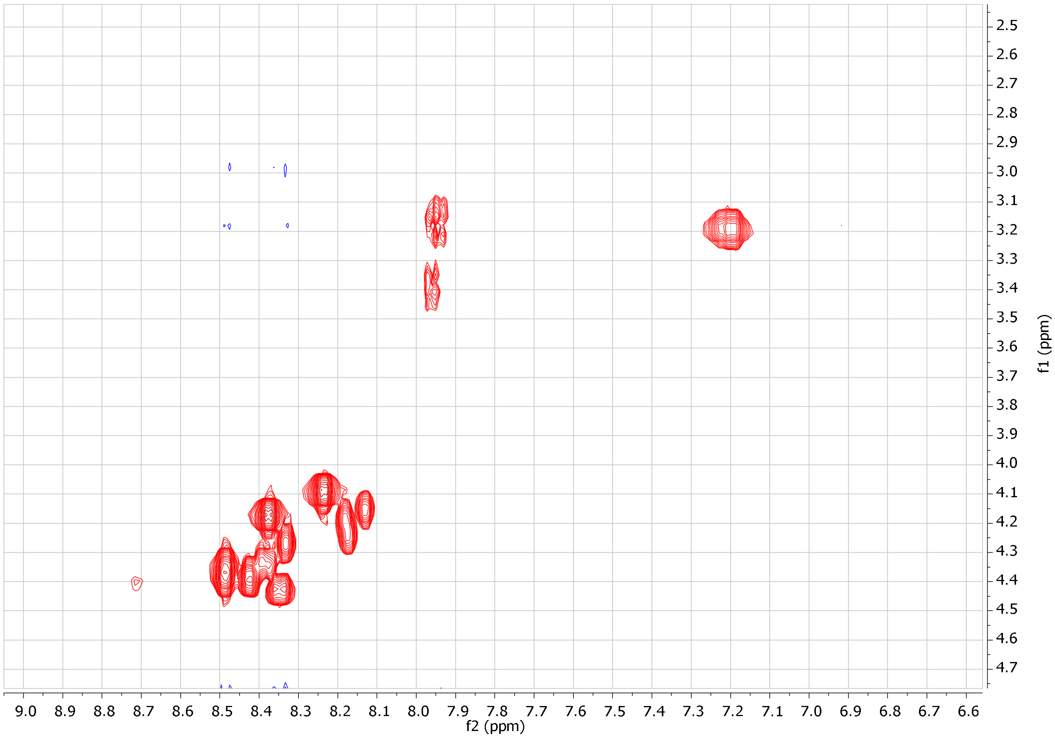

Figure S8. 2D ¹H COSY, ¹HN-¹Hα fingerprint region

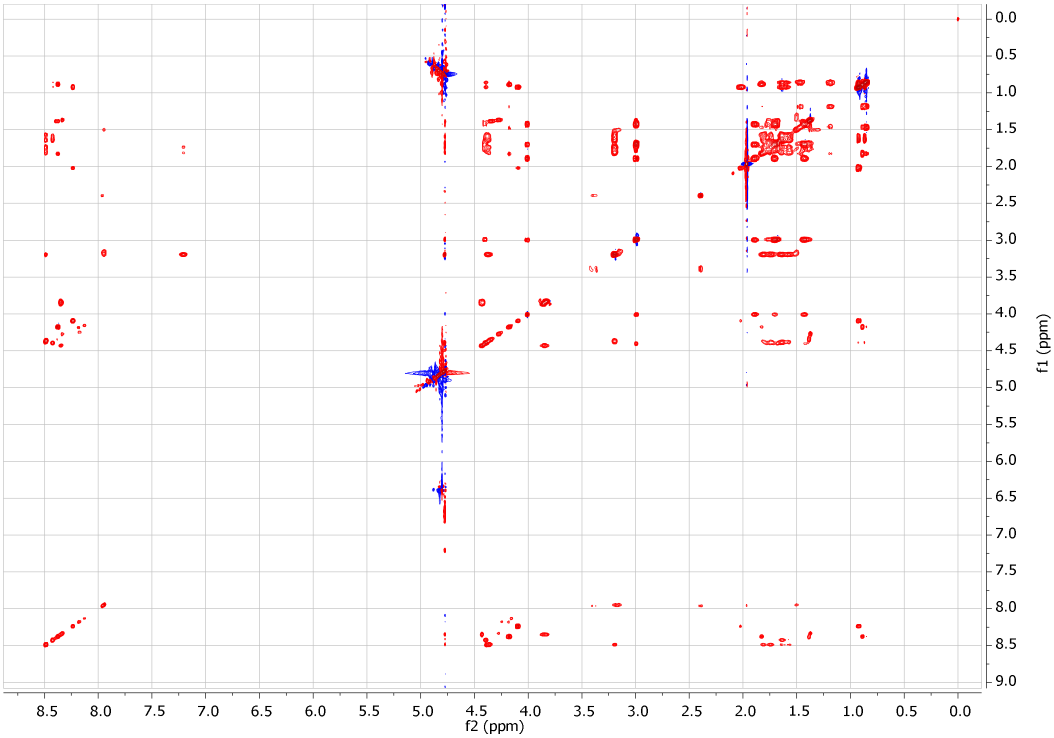

Figure S9. 2D ¹H TOCSY with 80 ms mixing time

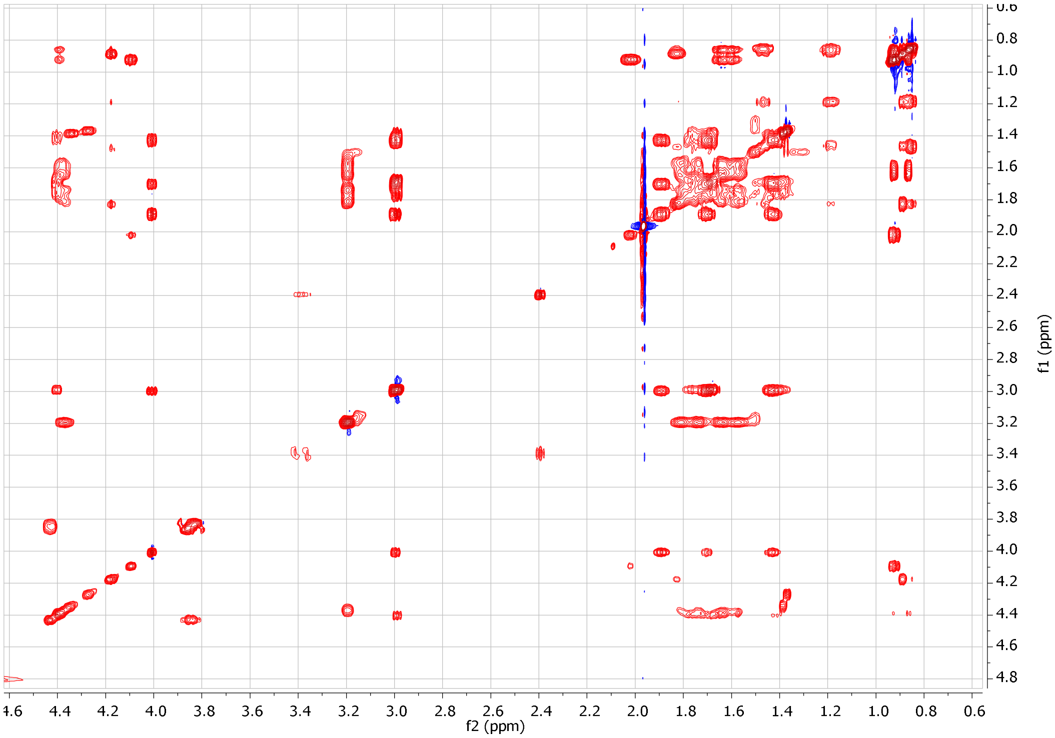

Figure S10. 2D ^1H TOCSY with 80 ms mixing time, aliphatic region

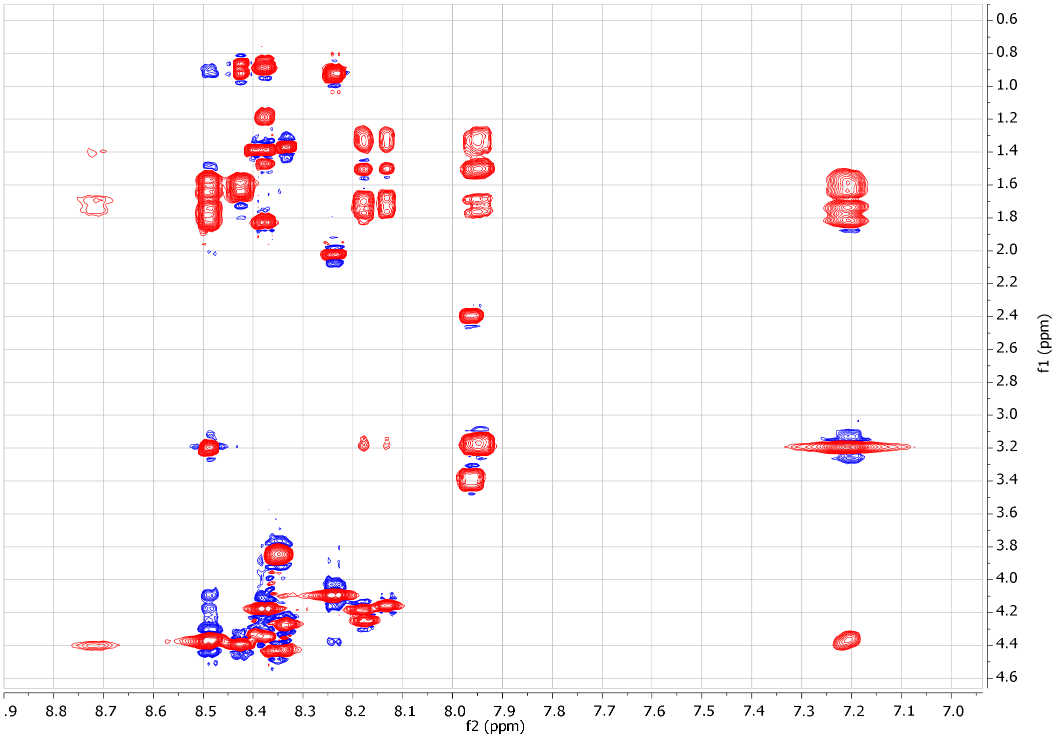

Figure S11. 2D ^1H TOCSY with 80 ms mixing time, ^1HN region

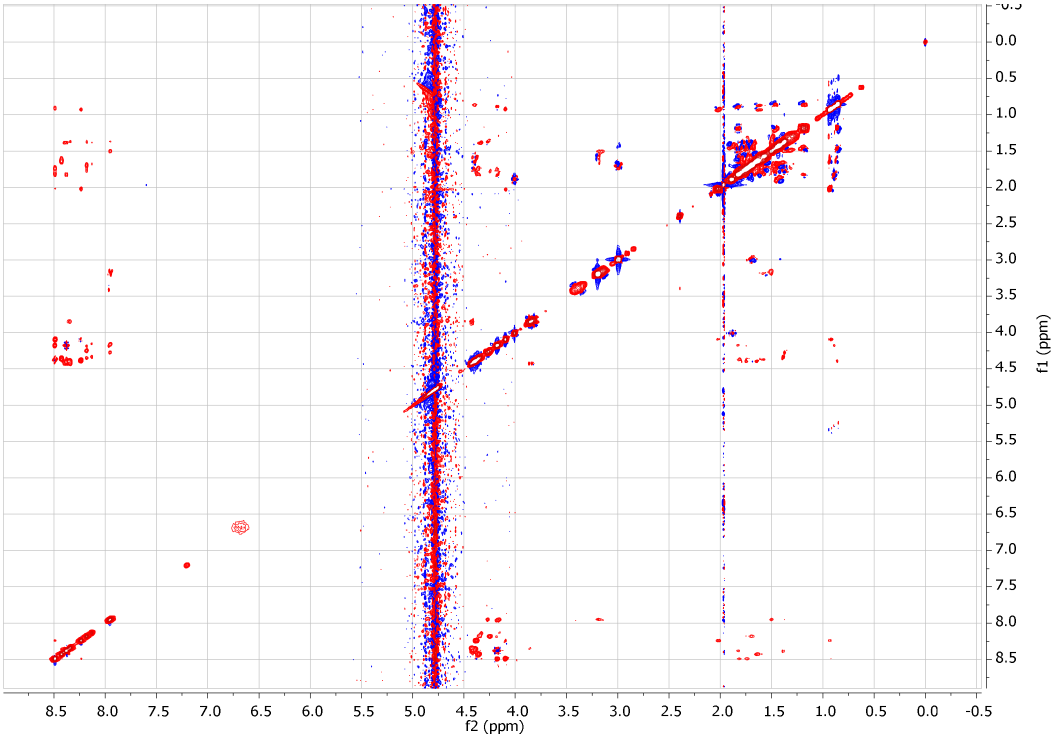

Figure S12. 2D ^{1}H NOESY with 100 ms mixing time

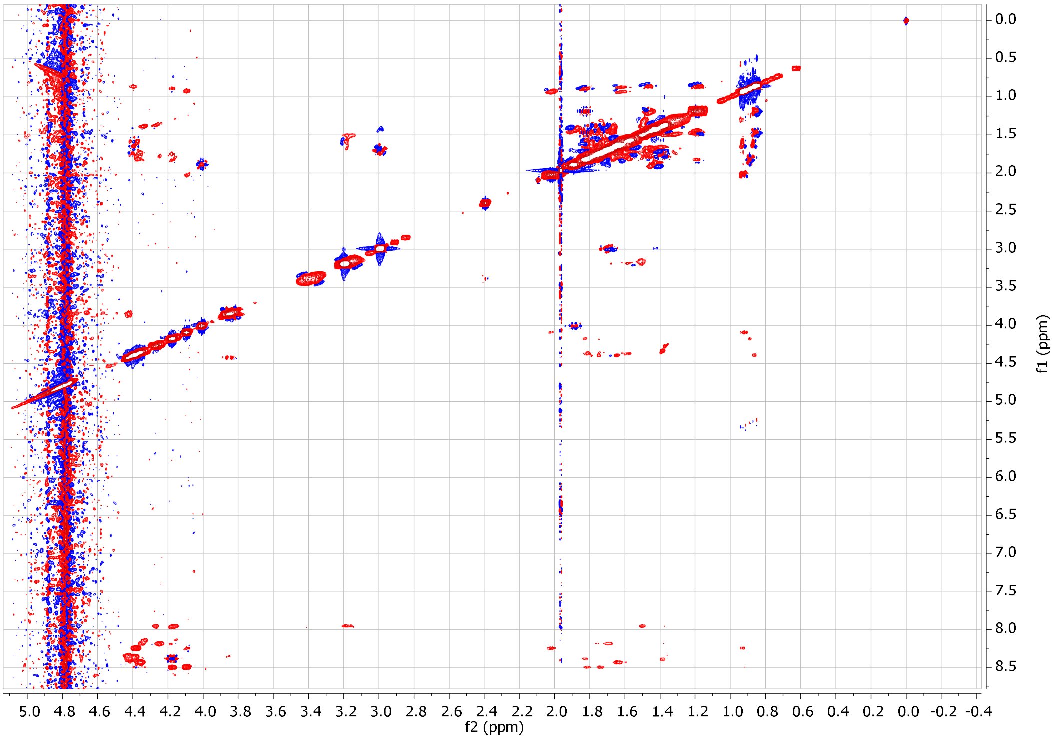

Figure S13. 2D ^{1}H NOESY with 100 ms mixing time, aliphatic region

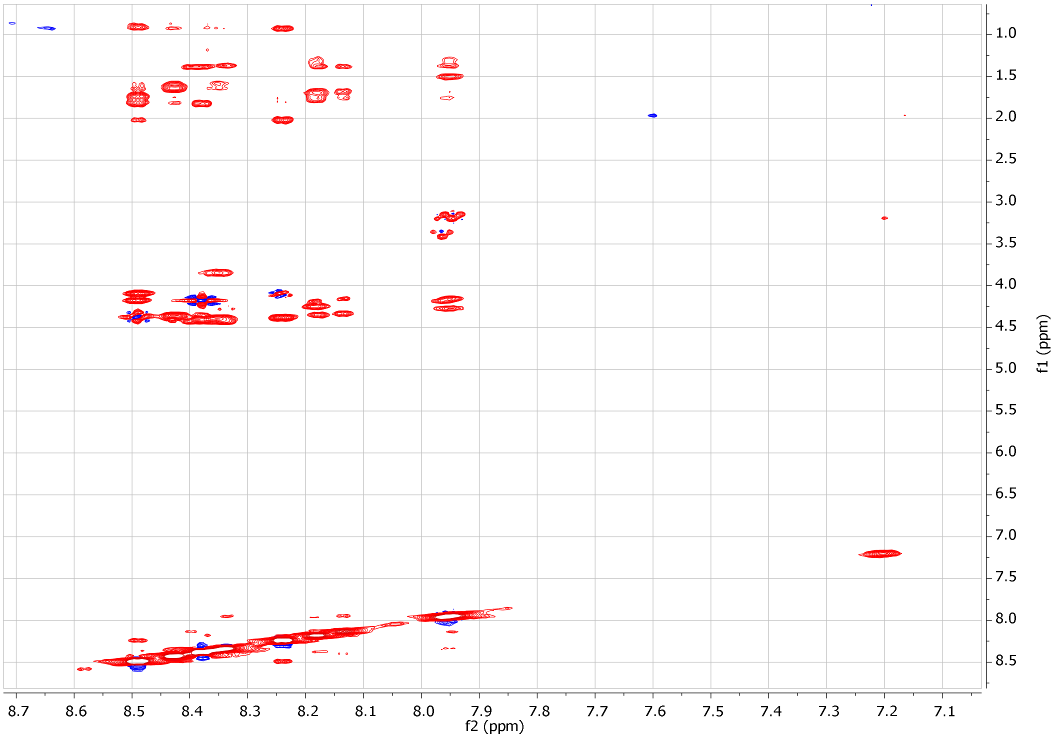

Figure S14. 2D ¹H NOESY with 100 ms mixing time, ¹HN region

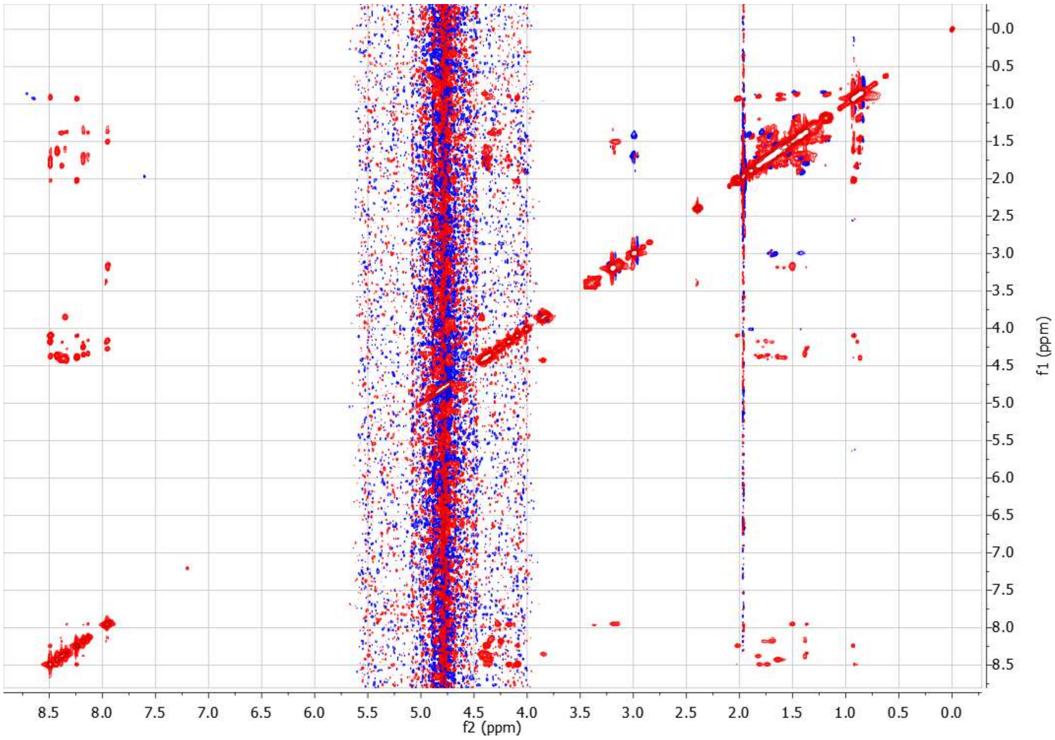

Figure S15. 2D ¹H NOESY with 200 ms mixing time

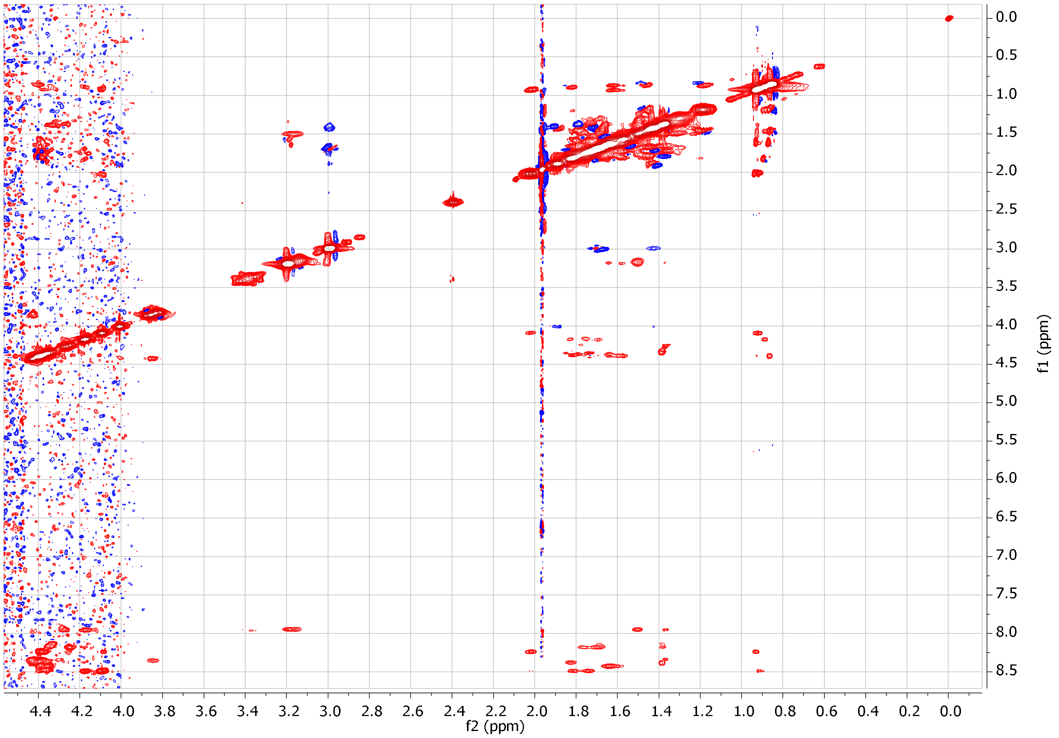

Figure S16. 2D ^{1}H NOESY with 200 ms mixing time, aliphatic region

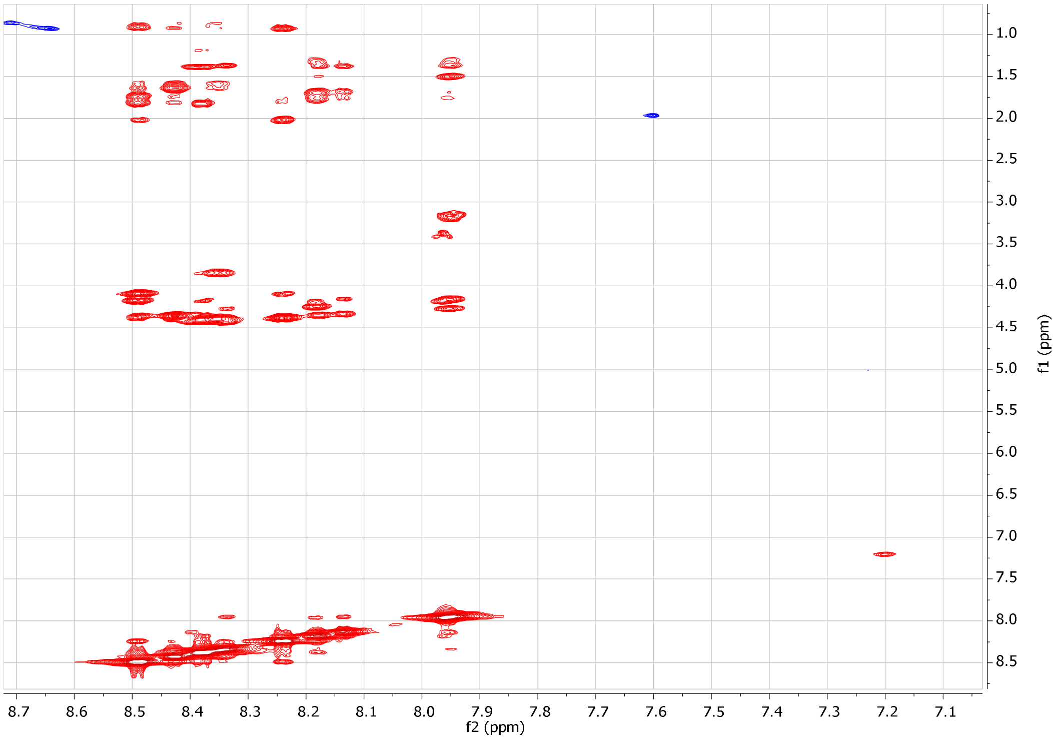

Figure S17. 2D ^{1}H NOESY with 200 ms mixing time, ^{1}HN region

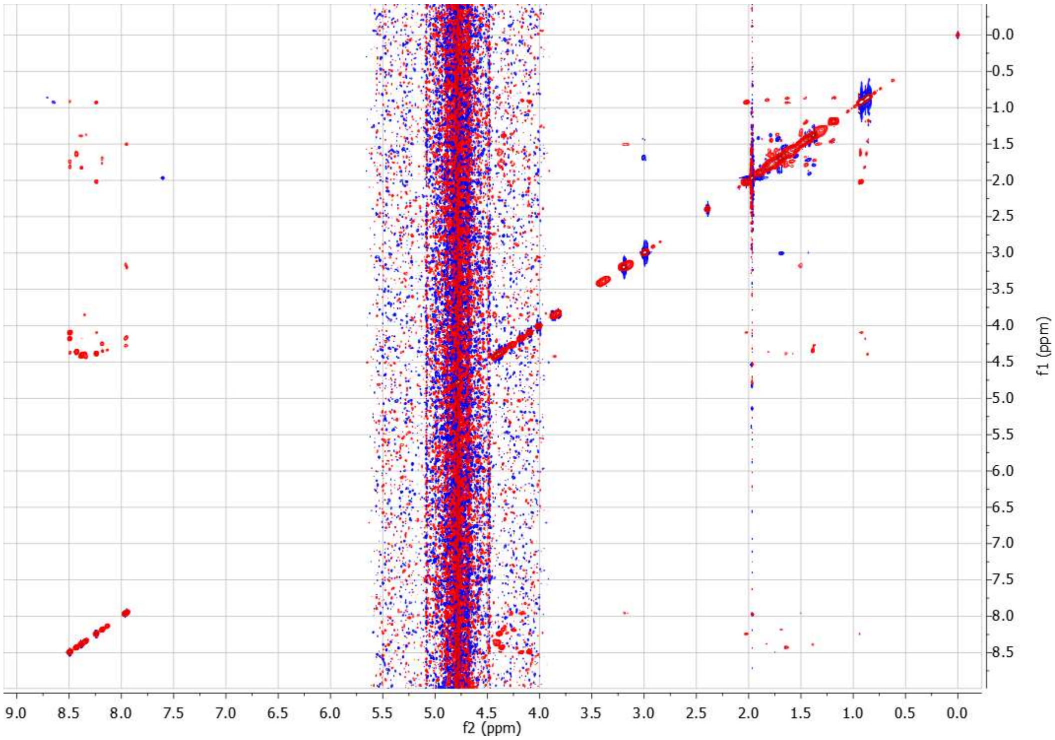

Figure S18. 2D ^{1}H NOESY with 400 ms mixing time

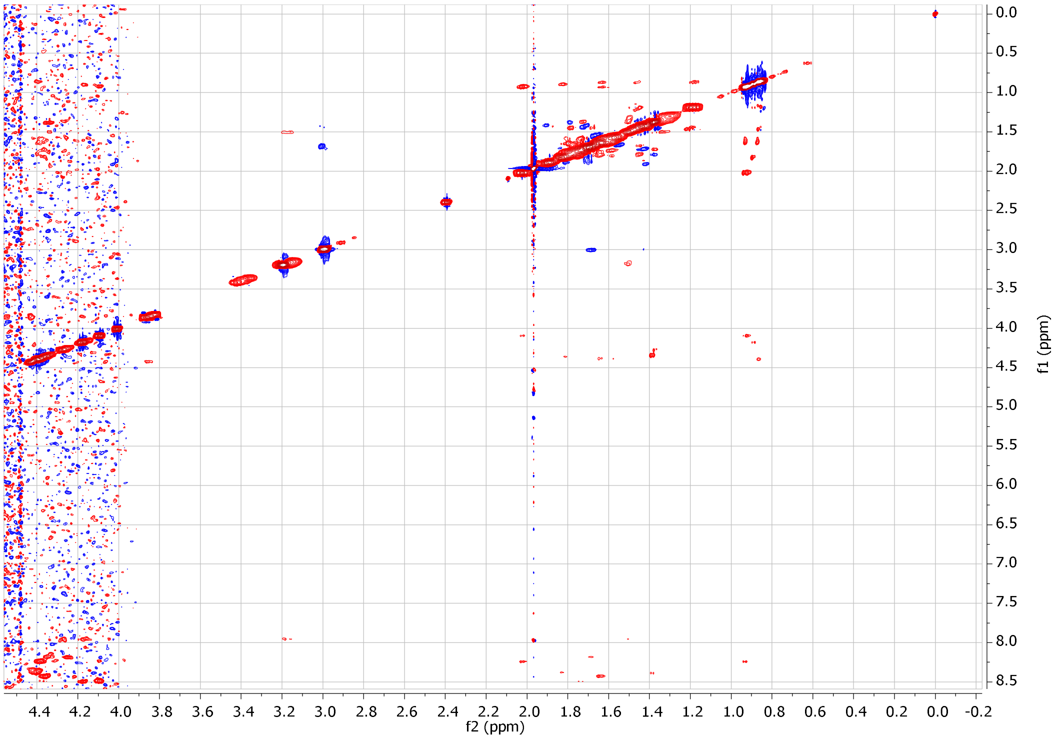

Figure S19. 2D ^{1}H NOESY with 400 ms mixing time, aliphatic region

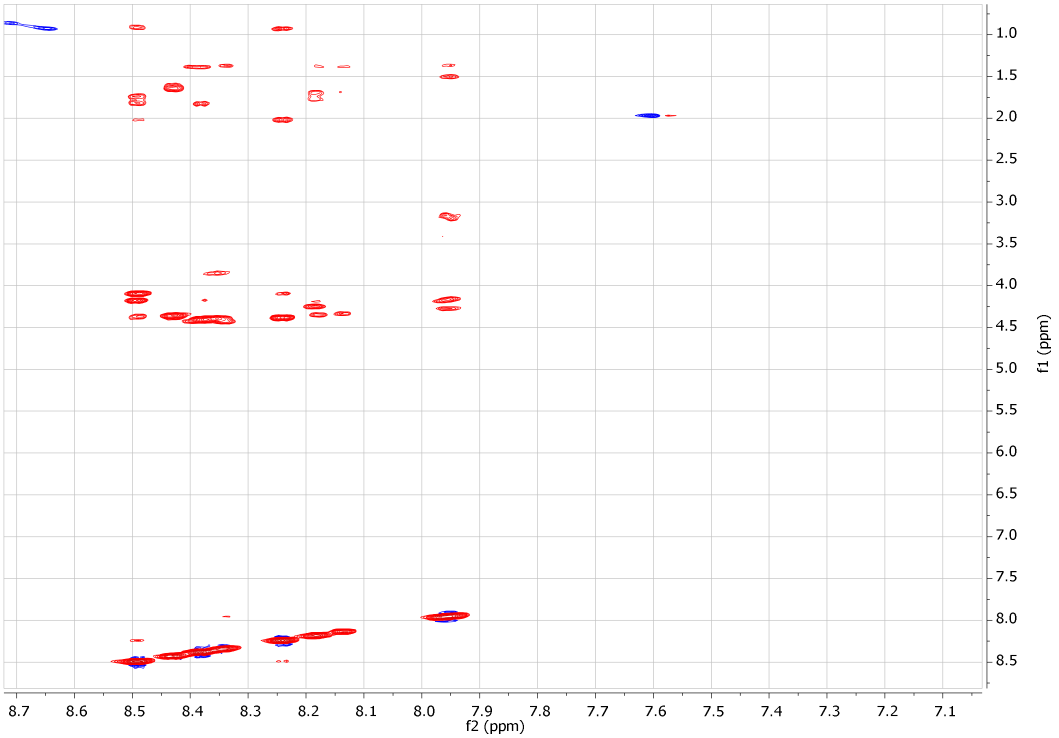

Figure S20. 2D ^{1}H NOESY with 400 ms mixing time, ^{1}HN region

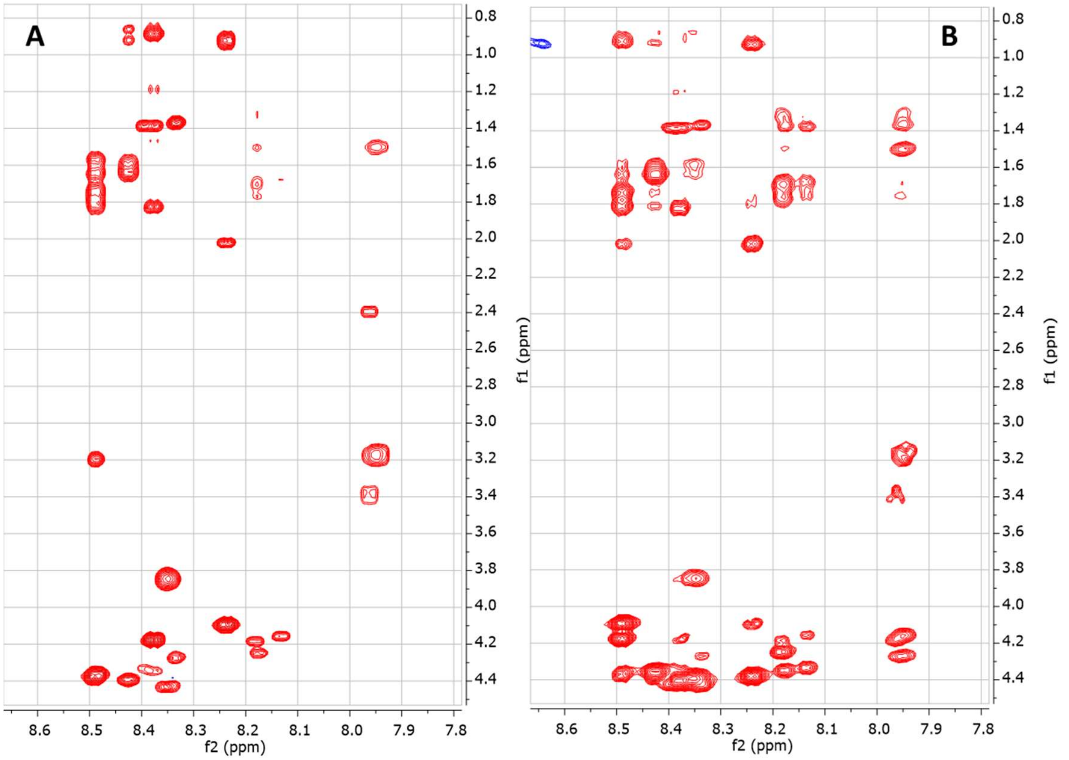

Figure S21. A) 2D ^{1}H TOCSY with 80 ms mixing time; **B)** 2D ^{1}H NOESY with 200 ms mixing time
